# Opium addiction as new source of lead poisoning: An emerging epidemic in Iran

**DOI:** 10.17179/excli2018-1355

**Published:** 2018-06-04

**Authors:** Samaneh Nakhaee, Omid Mehrpour

**Affiliations:** 1 Medical Toxicology and Drug Abuse Research Center (MTDRC), Birjand University of Medical Sciences, Birjand, Iran

## ⁯

Dear Editor,

From some years ago in Iran, there were frequent reports of hospitalized patients with lead poisoning. Interestingly, a large number of these reports were opium addicted patients. This experience proposed a new source of lead contamination.

Currently, lead (Pb) toxicity caused by Pb adulterated opium is a major health problem in Iran. The number of reported cases relating to Pb poisoning among opium users shows an upward trend in Iran. This issue reveals a new public health concern in Iran, a principal neighbor of Afghanistan, the major grower of opium worldwide (Alinejad et al., 2018[2]). 

Most cases of this non-occupational Pb poisoning (LP) were reported in Iran after 2004. The blood lead levels (BLL) of patients reported post 2010 show an increase compared to previous years (Alinejad et al., 2018[2]). Again, recent literature (2015-2018) suggests that there may be a current epidemic of LP among opium users in Iran. Reports of LP in opium users arise in peaks and troughs over the years in several different regions of Iran. For instance, in Tehran, from February 2016 to August 2017, 4294 lead poisoning cases were evaluated. Among them 80 cases were admitted with a BLL ranging from 47 to 1,124 μg/dL (Ghane et al., 2018[5]). In Kerman, between January and June 2016, a mean BLL:91.02 ± 59.83 μg/dL, range of 26 to 350 μg/dL (n=249) was reported (Hayatbakhsh et al., 2017[6]). Other reports from different regions include in Ardabil (April and May 2016, n=17, mean BLL: 93.36 ± 27.84 μg/dL, range: 48.4-144 μg/dL) (Farzaneh et al., 2017[4]), and Tehran (September 2015 - October 2016, n=119, mean BLL 76.2 fL (range 20 -316 fL) (Sadeghi et al., 2017[9]). In addition, more recent case reports from Tehran and Sari respectively show Pb toxicity in opium users (2017, BLL: 151 μg/ dL) (Zamani et al., 2017[13]) and (December 2016, BLL: 101.3 μg/dl) (Abedini and Karimi, 2017[1]). In Birjand from 2016, 1115 cases of lead poisoning were evaluated. Among them a sample of 69 opium users were admitted with signs and symptoms of LP accompanied by a mean BLL of 96.68 ± 48.65 μg/dL, ranging from 19.8 μg/dL to 402 μg/dL. In addition, there are unpublished reports from other cities such as Esfahan, Shiraz, Mashhad, and Mazandaran. Reports from other parts of the world include that of three Iranian people admitted to a Dutch hospital with BLLs ranging from 124.2 μg/dL to 223.6 μg/dL (van't Klooster et al., 2017[11]). In summary, approximately 4753 cases of LP, the majority of which were severe, were reported in opium users in Iran during 2015-2018. Although all lead poisoning cases in Iran is estimated more.

An interesting and alarming point in Ghane's report is maximum reported range for BLL (range: 47-1,124 μg/dL) (Ghane et al., 2018[5]). A number of other studies reported extremely high BLL values in opium users (Alinejad et al., 2018[2]; Soltaninejad and Shadnia, 2018[10]). BLL ≥ 100 µg/dL can induce cerebral edema, encephalopathy and death (Dooyema et al., 2012[3]; VanArsdale et al., 2004[12]) revealing the potential severity of the health hazard associated with adulterated opium.

The most widely accepted theories for the etiology of the contamination of opium with Pb are either for increasing its weight, at the production or at a local level; or for cosmetic reasons, to increase the appearance of quality or the addition of other products known to contain Pb such as Indian hair colouring; adulteration from the equipment used during processing and production of opium; and/or Pb contamination of the soil where opium is grown (Alinejad et al., 2018[2]; Hayatbakhsh et al., 2017[6]).

Regular users of opium can be considered as a vulnerable subgroup of the population in LP for a variety of reasons. It should be noted that are several common symptoms between Pb toxicity and opium addiction (Alinejad et al., 2018[2]). To the best of our knowledge, the most frequent complaints in LP include abdominal pain, arthralgia and myalgia (Karri et al., 2008[7]). Patients may therefore consume more opium in an effort to control the pain, unknowingly caused by LP, resulting in further Pb absorption; thus a vicious cycle develops. Additionally, it is likely that symptoms of LP in opium users may resemble withdrawal symptoms, potentially prompting increased opium use and subsequent Pb absorption (Meybodi et al., 2012[8]). Opioids decrease the bowel motility, potentially leading to further Pb absorption by prolonged exposure to the intestinal surface area, consequently resulting in an increased level of Pb in the bloodstream (Hayatbakhsh et al., 2017[6]). In Iran, opium is regarded as a privilege of the elderly, a population that are already more susceptible to inactivity and constipation. It can be reasonably stated that in the GI tract, opium's adverse effects are strengthened by Pb absorption and perpetuate a vicious cycle (Alinejad et al., 2018[2]). Older people are exposed to more Pb over their lifetimes. Pb gradually precipitates in the bones and this accumulated Pb can be an internal source of Pb in the blood in certain conditions such as aging and osteoporosis, where bone is reabsorbed.

Another factor to consider is diet; in Iran, most of Pb toxicity is due to opioids oral consumption, during fasting period. Absorption of Pb can be increased due to low calcium and iron in the diet or upon opium consumption while fasting (Alinejad et al., 2018[2]).

According to these data, it is very important that healthcare providers, not only those involved with drug abuse treatment, but all physicians, be aware of the potential for LP among patients with history of opium use, and the risks if undiagnosed.

## Acknowledgements

The authors want to convey an appreciation of Mrs. Elvira Conlon for nice comments in editing the manuscript.
